# Protective antioxidant effects of saffron extract on retinas of streptozotocin-induced diabetic rats

**DOI:** 10.22336/rjo.2020.61

**Published:** 2020

**Authors:** Georgios Skourtis, Anthi Krontira, Stavroula Ntaoula, Anastasia Varvara Ferlemi, Konstantina Zeliou, Constantinos Georgakopoulos, Georgios Marigoula Margarity, Nikolaos Fotini Lamari, Nikolaos Pharmakakis

**Affiliations:** * Ophthalmology Clinic, Department of Medicine, University of Patras, Rio, Greece; ** Laboratory of Human and Animal Physiology, Department of Biology, University of Patras, Patras, Greece; *** Laboratory of Pharmacognosy & Chemistry of Natural Products, Department of Pharmacy, University of Patras, Rio, Greece

**Keywords:** diabetes mellitus, saffron, streptozotocin, retina, antioxidant defense enzymes, lipid peroxidation

## Abstract

**Objective:** Oxidative stress plays an important role in the pathogenesis of diabetic retinopathy. The aim of the present study was to investigate the effect of Crocus sativus L. styles (saffron) extract on oxidative stress indices of retina in streptozotocin (STZ)-induced diabetic rats.

**Methods:** Adult male Wistar rats (n=20) were randomized into the following 4 groups (n=6-7/ group): Control group (C): normal, Control + Saffron group (CS): non-diabetic rats treated with 60 mg/ kg of saffron extract, Diabetic group (D) and Diabetic + Saffron group (DS): diabetic rats treated with 60 mg/ kg saffron extract. We determined the activity of superoxide dismutase (SOD), glutathione peroxidase (GPx) and catalase (CAT) as markers of antioxidant response, as well as malondialdehyde (MDA) as a marker of lipid peroxidation.

**Results:** Induction of diabetes caused a significant decline in the activities of CAT (76.43%), SOD (53.43%) and GPx (77.58%). MDA levels were significantly lower in the DS group (0.878 ± 0.375 nmol MDA/ mg protein) as compared to D group (1.950 ± 0.299 nmol MDA/ mg protein, p<0.01) and in the CS group (0.503 ± 0.221) in comparison to C group (1.699 ± 0.454, p<0.01). Moreover, SOD and GPx activities were significantly higher (more than 1.5 and 3.5-fold respectively) after treatment with saffron (p<0.01). Regarding the retinas of non-diabetic animals, the administration of the extract caused an > 1.8-fold increase in the activity of CAT (p<0.05) and a 3-fold decrease in MDA levels (p<0.01).

**Conclusions:** This study showed that saffron extract has a protective antioxidant action in retinas of diabetic rats.

**Abbreviations:** C = Control group, CS = non-diabetic rats diabetic rats treated with 60 mg/ kg saffron extract, D = diabetic group, DS = diabetic rats treated with 60 mg/ kg saffron extract, SOD = superoxide dismutase, GPx = glutathione peroxidase, CAT = catalase, MDA = malondialdehyde, DM = diabetes mellitus, DR = diabetic retinopathy, ROS = reactive oxygen species, STZ = streptozotocin, GSH = reduced glutathione

## Introduction

Diabetes mellitus (DM), a highly prevalent and heterogeneous metabolic disorder, is characterized by dysregulation of insulin secretion and insulin action leading to disturbances in basal metabolism and chronic hyperglycemia, the key hallmark of the disease [ **1**]. Chronic uncontrolled hyperglycemia is regarded as risk factor for secondary complications of the cardiovascular, renal, neurological, and ocular systems [ **2**, **3**]. Diabetic retinopathy (DR) is recognized as one of the most common afflictions of the small vessels of the retina due to uncontrollable high concentration of glucose and a dominant reason for non-hereditary sightlessness among the humans of occupational age [ **3**]. Despite the fact that the exact procedure of the progression of complications of diabetes has not been established yet, many studies demonstrate that oxidative stress, which is the imbalance between the antioxidant mechanisms and the reactive oxygen species (ROS), is involved in every possible causal process [ **4**, **5**], like glucose autoxidation, polyol pathway, prostanoid synthesis, protein glycation, PKC activation, and the hexosamine pathway [ **4**, **6**].

In regular metabolic conditions, ROS are scavenged by enzymes, such as superoxide dismutase (SOD), catalase (CAT) and glutathione peroxidase (GPx). Chronic hyperglycemia provokes augmentation of ROS and reduced activity of antioxidant enzymes bringing about disorders like DR [ **4**, **5**, **7**, **8**]. Long-standing oxidative stress damages biomacromolecules such as nucleic acids, lipids, proteins and induces lipid peroxidation in membranes [ **4**], leading to the accumulation of toxic malondialdehyde (MDA). Impairments in mitochondrial DNA are caused by ROS, which lead to faults in transcription of electron transport chain components and finally worsen ROS generation [ **9**]. Moreover, lipid membrane leakage in mitochondria results in apoptosis in pericytes and endothelial cells [ **10**]. Retinal cells contain large quantities of membrane polyunsaturated fatty acids and present the highest oxygen consumption and glucose oxidation among various tissues. Moreover, lipid membrane lipid leakage in mitochondria results in apoptosis in pericytes and endothelial cells [ **9**]. Therefore, retina is considered sensitive to oxidative stress [ **11**]. Oxidative changes have been detected in the initial stages of DR, and are not efficiently corrected by establishing regulation of glucose [ **12**]. Metabolic adjustments are difficult to be consistently attained in diabetic subjects. Therefore, alternative solutions are necessary so that eye complications in diabetes can be arrested [ **13**]. Natural antioxidants have shown effectiveness concerning the progression of the first stages of diabetic retinopathy [ **14**]. Treatment with green tea and curcumin ameliorated the activity of SOD and CAT in retinas of diabetic rats and protected retinal capillaries against decrease of basement membrane thickness [ **15**, **16**]. Arnal et al. proved that administration of lutein and docosahexaenoic acid normalized MDA levels and GPx activity in retinal tissues of animals with diabetes and protected the ganglion cells number and the cells in outer and inner nuclear layers [ **17**].

The dried styles of *Crocus sativus* L (family *Iridiceae*) constitute saffron and are rich in apocarotenoids. Saffron has been used traditionally in the therapy of various disorders like cough, asthma, menstruation issues, insomnia, pain, colic, chronic uterine hemorrhage, cardiovascular problems and cancer [ **18**]. Accordingly, pharmacological studies have demonstrated that saffron possesses anti-anxiety [ **19**], anticonvulsant [ **20**], antidepressant [ **21**], anti-inflammatory [ **22**], antitumor [ **18**], hypotensive [ **23**], and anti-oxidant [ **24**- **27**] properties. Motamedrad et al. showed that the administration of saffron stigma extract improved plasma lipid peroxidation in an STZ experimental model of diabetes [ **28**]. Saffron administration brought about an improvement of antioxidant enzymes CAT, SOD and GPx and a decrease in the levels of MDA in streptozotocin-induced diabetes [ **29**]. Treatment with crocin (active constituent of saffron) provoked a decline in MDA levels and a rise in GPx activity in hippocampus and cerebral cortex of rats with STZ-induced oxidative stress [ **30**]. Bandegi et al. showed that treatment with saffron and crocin improved oxidative stress indices in chronic stress induced oxidative damage in brain, liver and kidney [ **31**]. Similar results regarding the activities of antioxidant enzymes SOD, CAT and GPx were reduced by Samarghandian et al. in the hippocampus of rats with diabetic encephalopathy treated with saffron [ **32**]. Moreover, regarding retina, saffron has been found to alleviate circulatory system, improve macular disease and ischemia caused by aging [ **33**]. In a recent clinical study, Sepahi et al. indicated a beneficial effect of oral crocin in diabetic macular edema [ **34**]. There are also numerous clinical studies proving effect of saffron in age related macular degeneration regarding mainly visual acuity and amplitude of fERG [ **35**]. Fernández-Sánchez et al. determined that safranal treatment in experimental retinitis pigmentosa had a beneficial effect against the decrease of photoreceptors and slowed the degeneration of inner layers of retina and its capillary network [ **36**]. Crocetin, another active constituent of saffron, was found to have antiischemic and antioxidant actions in a retinal ischemic model in mice [ **37**].

Furthermore, saffron has shown antidiabetic activity and concomitant treatment with saffron and insulin seems to improve insulin sensitivity [ **38**]. In addition, saffron is regarded to be safe for medical use in patients since the median lethal dose of intraperitoneal administration of ethanolic saffron extract in rats is 3.500 g/ kg, while renal and hepatic dysfunction is caused by doses over 0.350 g/ kg for 14 days [ **39**], that is regarded as equivalent to 0.056 g/k g/ day for human [ **40**]. To the best of our knowledge, no previous study has evaluated the antioxidant status in diabetic retinopathy after administration of saffron. Therefore, in the present study, we investigated the potential effect of *Crocus sativus* L. styles (saffron) extract on redox indices of retina tissue in streptozotocin (STZ)-induced diabetic rats. The SOD, GSH-Px, CAT activities were determined as markers of antioxidant defense and MDA levels and as product of lipid peroxidation [ **41**].

## Methods

**Animals**

Diabetes was induced in adult (10-14-week-old) healthy male Wistar rats with a single intraperitoneal injection of streptozotocin (55 mg/ kg in freshly prepared 0.05 M sodium citrate buffer, pH 4.5, day 0) [ **42**, **43**]. Age-matched non-diabetic rats, which were injected with the same amount of normal saline buffer, served as controls. Blood glucose was measured prior to the induction of diabetes and 48 h after streptozotocin/ vehicle injection in all groups. Rats with glucose-serum levels greater than 250 mg/ dL 48 hours post-injection [ **43**], as determined by the Contour glucose commercial kit (Bayer), were considered diabetic. The animals were kept in stainless-steel cages in adequately aired rooms with stable temperature (23°C) and humidness conditions and 12 h: 12 h light-dark periods. The rats were free regarding the uptake of food (typical laboratory animal nutrition consisted of dry pellets) and drinking water. Weight and fasting glucose were estimated every 2 weeks during the experimental period to regulate the dosage of the herbal extract in accordance with body weight of the rats and to evaluate their glycemic status. The investigation was complied with the Association for Research in Vision and Ophthalmology Statement for Use of Animals in Ophthalmic and Vision Research and Guiding Principles in the Care and Use of Animals [ **44**]. The research has been sanctioned by the University Hospital Bioethics Committee.

**Experimental design**

Animals were randomized into the following four groups (n = 5/ group):

- Control group (C): non-diabetic rats, which received only intraperitoneal injection of normal saline buffer every other day;

- Control + Saffron group (CS), which received *Crocus sativus* extract (60 mg/ kg) every other day;

- Diabetic group (D), which were administered only intraperitoneal injection of saline buffer every other day; 

- Diabetic + Saffron group (DS), treated with *Crocus sativus* extract (60 mg/ kg) every other day.

The treatment started 2 weeks after the injection of streptozotocin and lasted for 10 weeks. There was an adaptation of the dose every two weeks taking into account the alterations in body weight. One or two units of insulin was administered subcutaneously two or three times per week, taking into consideration the glycemic state of the rats [ **42**]. At the end of week 12, the rats were sacrificed by overdose of pentobarbital (100 mg/ kg). Eyes were removed and retinas were immediately isolated.

**Chemicals**

Commercially available saffron styles were obtained from COOPERATIVE DE SAFRAN/ CROCOS-50010 KOZANI and saline from local drugstore. Methanol (HPLC grade) was purchased from Chem-Lab NV (Zedelgem, Belgium), ultrapure water (18 MOhm*cm) was produced by a Milli-Q system (Merck Millipore, USA). Citric acid (>99 %) and trisodium citrate trihydrate (>99%) were purchased from Merck (Darmstad, Germany). Streptozotocin, catalase (CAT) from bovine liver (4966 U/ mg), glutathione peroxidase (GPx), Bradford reagent and bovine serum albumin were purchased from Sigma-Aldrich (St. Louis, MO). SOD activity was determined by using a Superoxide Dismutase Assay Kit from Cayman Chemical Company (Ann Arbor, USA). 

**Preparation of saffron extract**

Thirty grams of saffron styles powder was extracted with 2 L aqueous methanol (methanol: water 50% v/ v) for 24 h at room temperature), with continuous stirring in the absence of light. The extract was centrifuged at 3000g for 15 min and filtered under vacuum through cellulose filter. Methanol was evaporated and the residual liquid was lyophilized using freeze drying system (Labconco Corp., Kansas City, MO). Analysis was performed with high-performance liquid chromatography (HPLC) and it was the same as previously reported [ **24**, **45**]. The dry extract was stored at -20°C until further use. Final samples were prepared by dissolving 1 g of dry extract in 72 mL saline (18 mL/ 250 mg), filtered under vacuum through membrane filter (0.45 μm) and sterilized through second membrane filtration (0.2 μm i.d.).

**Tissue treatment**

After dissection, the tissues were rinsed in icy saline solution to eliminate blood and then weight estimation was conducted. The samples were homogenized in 30 mM phosphate buffer, pH 7.6 (10% w/ v), and centrifuged at 12,600 × g for 20 min at 4°C. The homogenates were kept frozen at -80°C, pending further biochemical analysis.

**Assessment of antioxidant parameters**

Assay of superoxide dismutase activity 

SOD activity was measured by the Superoxide Dismutase Assay Kit. A tetrazolium salt was formed to identify superoxide radicals produced by xanthine oxidase and hypo¬xanthine. One unit of SOD activity is described as the quantity of enzyme that dismutates superoxide radical by 50%.

**Assay of catalase activity**

Activity of CAT was evaluated by using the modified method of Sinha [ **46**]. It is measured in μmol/ min/ mg of protein.

**Glutathione peroxidase activity**

GPx activity was estimated according to Rotruck et al. [ **47**] to estimate the rate of the glutathione oxidation by H2O2. One unit of GPx activity per minute is defined as the amount of enzyme needed for the conversion of 1 μmol of reduced glutathione to the oxidized form of it.

**Malondialdehyde levels**

Lipid peroxidation was estimated by measuring MDA levels through the fluorometric method of Grotto et al. [ **48**]. The results were expressed as nmol MDA/ mg tissue.

**Statistical analysis**

All estimations were done in triplicates and results were expressed as mean ± standard deviation. Before statistical analysis, all variables were tested for normality and homogeneity of variance by using the Kolmogorov-Smirnoff and Levene tests, respectively. One-way analysis of variance was performed to compare biochemical parameters among the four groups followed by Bonferroni and Games-Howell corrections (the last was performed when the homogeneity of variance was violated). Mann Whitney-U was performed where appropriate. P<0.05 was considered to be statistically significant. The analysis was performed using IBM SPSS Statistics 19.0 software (SPSS, Inc., Chicago, IL). 

## Results

*Body weight and Blood Glucose*

**Fig. 1:**
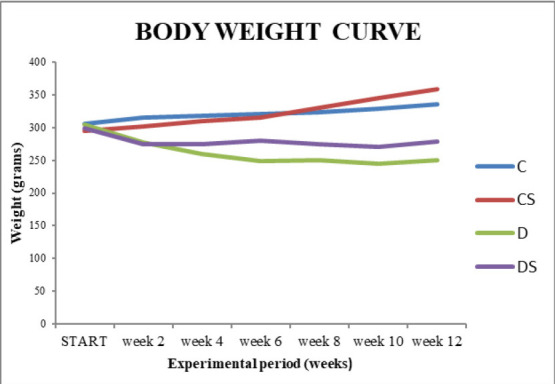
Body weight curve during the experimental time of 12 weeks. C (Control), CS (Control, treated with Saffron), D (Diabetic), DS (Diabetic treated with Saffron)

A significant deterioration of hyperglycemia, assessed by changes in body weight and blood glucose was noted in rats in the D group in comparison with the C group. Body weight and blood glucose, measured for last time before euthanasia, were comparable in the diabetic groups (D and DS) and were significantly different (P<0.001 regarding weight and P<0.01 regarding glucose) from those in the non-diabetic control group ( **Table 1**, **Fig. 1**). A slight increase in body weight was observed in animals that received saffron.

**Table 1:** Effect of saffron extract on Body weight and Glucose in Rats conducted in June 2019

Group	Bodyweight (g)	Glucose (mg/ dL)
C	336.00 ± 10.68	75.00 ± 11.55
CS	358.40 ± 19.97	87.20 ± 8.87
D	250.40 ± 20.32*	447.20 ± 66.60†
DS	278.40 ± 24.80	456.73 ± 42.58
Data is mean ± SD, n ═ 5 C (Control), CS (Control, treated with Saffron), D (Diabetic), DS (Diabetic treated with Saffron), SD (standard deviation). Different symbols express statistically significant difference between groups. *p<0.001 compared with group C, †p<0.01 compared with group C.		

*Antioxidant parameters*

**Fig. 2:**
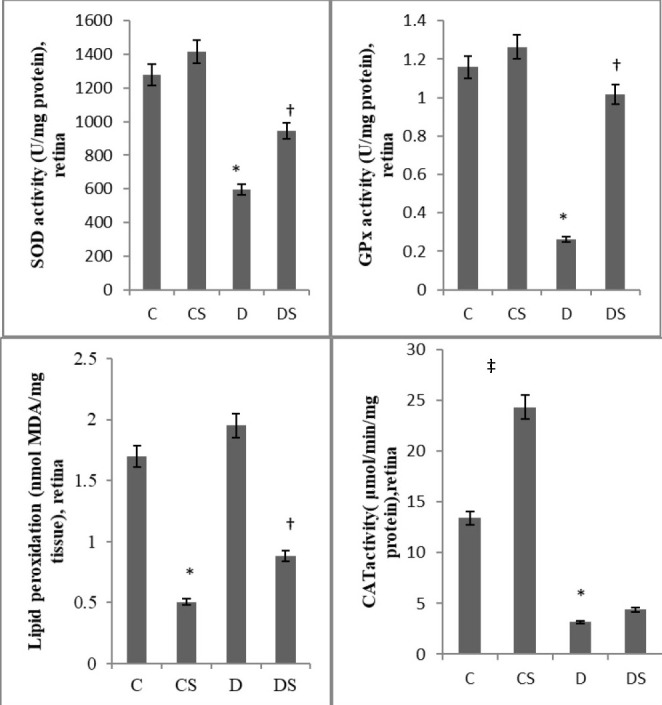
Effect of saffron extract on Antioxidant Parameters and Lipid peroxidation in Rat Retina. Data is mean ± SD, n ═ 5. C (Control), CS (Control, treated with Saffron), D (Diabetic), DS (Diabetic treated with Saffron), SOD (Superoxide dismutase), GPx (Glutathione Peroxidase), CAT (Catalase), MDA (Malondialdehyde), SD (standard deviation), *p<0.01 compared with group C, ‡p<0.05 compared with group C, †p<0.01 compared with group D

The impact of the administration of *Crocus sativus extract* in the antioxidant status in the retina was estimated by the determination of the activities of enzymes SOD, CAT and GPx. SOD activity (units/ mg protein) in the retinas of the diabetic rats was less than half of the retinas of control rats (595.28 ± 149.49 versus 1278.18 ± 296.21, P<0.01). Administration of saffron extract rescued to some extent the diabetes-induced decrease of SOD, taking into account that the activity of the enzyme in DS group was 1.59-fold higher (944.19 ± 279.38) than in D group (P<0.01). Likewise, the levels of GPx (U/ mg protein) (0.26 ± 0.11, ↓77.58%) and CAT (μM/ min/ mg of protein) activity (3.16 ± 0.96, ↓76.43%) in retinas of the animals from group D when compared with those in the retinas obtained from animals from group C (1.16 ± 0.42 and 13.41 ± 5.12 respectively), were significantly different. The administration of *Crocus sativus* extract also significantly prevented GPx activity reduction, (1.02 ± 0.54, equivalent to 3.9-fold greater activity in DS in relation to D group, P<0.01). In contrast to the beneficial effect of saffron, in the DS group, concerning SOD and GPx, treatment did not achieve a significant increase in CAT activity. Nevertheless, a statistically significant difference in CAT activity was recorded in Group CS (24.32 ± 9.66) in relation to Group C ( **Fig. 2**).

*Oxidative damage*

Oxidative damage was assessed as the levels of MDA (nmol MDA/ mg protein). There was an increase in lipid peroxidation in rat retina in D group as related to C (1.95 ± 0.30 versus 1.70 ± 0.45) but the increase was not significant. Administration of Crocus sativus extract induced a significant drop regarding MDA in both CS and DS groups (0.50 ± 0.22 and 0.88 ± 0.38 respectively, P<0.01) ( **Fig. 2**).

## Discussion

Retinal tissue is dense in polyunsaturated fatty acids, continuously exposed to light and has the highest consumption of oxygen and glucose oxidation compared to any other tissue. Therefore, retina is extremely sensitive to diabetes-related damage [ **11**, **49**]. Previous studies have demonstrated that long standing hyperglycaemia leads to increased production of ROS, chronic oxidative stress and decrease in activity of endogenous antioxidant defense enzymes responsible for scavenging free radicals, such as SOD, CAT, and GPx [ **4**, **5**, **7**, **8**]. Compromise of the activities of CAT and SOD can result in an increased amount of O2.- and H2O2, which in turn produce OH., leading to excessive lipid peroxidation, and the formation of toxic malondialdehyde (MDA). Furthermore, elevated lipid peroxidation impairs membrane function by diminishing the fluidness of membranes and changing the activity of membrane-bound enzymes and receptors, finally leading to impairment of biological components [ **50**]. SOD, the first enzyme in the chain of antioxidant defense, acts against further production of ROS through catalyzing the conversion of O2.- into H2O2, which is afterwards deactivated to H2O by CAT or GPx [ **51**]. CAT exerts its action through the disintegration of H2O2 to H2O and O2 and the protection against highly reactive OH. radicals [ **52**]. GPx converts H2O2 to H2O and O2 by using glutathione (GSH) as a proton donor [ **53**]. Glycation of SOD and CAT, which occurs in diabetes, or inactivation of SOD by H2O2, leads to a reduction in enzyme action [ **53**- **55**]. It has been demonstrated that subnormal GPx activity in diabetes could be attributed to the impacts of the raised quantities of radicals [ **56**] and the decreased availability of glutathione [ **57**].

We investigated the extent of possible antioxidant protection offered by saffron aqueous extract by measuring the activities of the above enzymes and the degree of lipid peroxidation in retinas of non-diabetic and diabetic rats. Saffron has been the objective of growing interest in ophthalmology recently and several experiments dealing with possible beneficial effects on eye have been conducted [ **34**, **35**, **58**- **61**]. According to Evans [ **62**], saffron extract contains ingredients with antioxidant actions [ **63**], which may alleviate decreased insulin secretion and impaired insulin resistance and protect tissues against diabetes complications. Our experimental procedures demonstrated a significant drop-in activity of antioxidant enzymes in retinas of rats with diabetes and an elevation in MDA levels, though not significant. MDA levels in rats that received Crocus sativus extract were also significantly reduced in both DS and CS groups. Moreover, a significant increase was recorded in the activities of GPx and SOD after the administration of the herbal extract. Regarding CAT activity, it was significantly higher in group CS in relation to group C although we did not record any important difference between D and DS. The alterations in the activities of antioxidant enzymes are compatible with previous investigations in which animals had been treated with saffron; parameters have been assessed in different tissues [ **58**, **64**- **66**]. More specifically, Makri et al. showed that the intraperitoneal administration of saffron extract protected lenses of newborn selenite-treated rats against lipid peroxidation and reduction of the activities of antioxidant parameters CAT, SOD and GPx [ **58**]. It can be suggested that the administration of saffron extract brought about an improvement in the status of oxidative stress in retina.

## Conclusion

To the best of our knowledge, this is the first study that investigated the effect of *Crocus sativus* styles extract on oxidative stress indices in a STZ animal experimental model of diabetic retinopathy and demonstrated significant beneficial changes in redox indices. Taking our results and previous experiments into consideration, it can be deducted that the administration of the specific dosage of saffron extract may have a role in restoring the action of antioxidant defensive enzymes and decreasing lipid peroxidation in diabetic retina. Nevertheless, many issues regarding the antihyperglycemic actions of *C. sativus* extract should be further examined. Therefore, new experiments, using different concentrations of saffron, its isolated compounds or combinations with other natural antioxidants, are needed before its introduction in diabetic retinopathy therapy. However, our outcomes proposed that *Crocus sativus* may be used as complementary treatment in diabetic retinopathy after randomized clinical trials.

**Acknowledgments**

All authors have substantial contribution to this paper.

**Sources of Funding**

None.

**Disclosures**

None.
